# RNA-Seq Analysis of Equine Conceptus Transcripts during Embryo Fixation and Capsule Disappearance

**DOI:** 10.1371/journal.pone.0114414

**Published:** 2014-12-16

**Authors:** Yurika Tachibana, Toshihiro Sakurai, Hanako Bai, Kunio Shiota, Yasuo Nambo, Kentaro Nagaoka, Kazuhiko Imakawa

**Affiliations:** 1 Laboratory of Theriogenology and Animal Breeding, Veterinary Medical Sciences, The University of Tokyo, Tokyo, 113–8657 Japan; 2 Laboratory of Cellular Biochemistry and Animal Resource Center, Veterinary Medical Sciences, The University of Tokyo, Tokyo, 113–8657 Japan; 3 Hidaka Training and Research Center, Japan Racing Association, Urakawa, 057–0171 Japan; 4 Laboratory of Veterinary Physiology, Department of Veterinary Medicine, Tokyo University of Agriculture and Technology, Fuchu, 183–8509 Japan; University of Missouri, United States of America

## Abstract

Extensive studies have been conducted to characterize the unique phenomena of equine pregnancy. Most studies have focused on embryo transmigration when the embryo is covered with a mucin-like glycoprotein capsule and on the characterization of the chorionic girdle and chorionic gonadotropin (CG) secretion. However, the events preceding and following capsule disappearance have not been well studied. In this study, the mRNA expression in conceptus membranes at days 19, 21, and 25 (day 0 = day of ovulation) was analyzed by RNA-seq (SOLiD3), and transcript levels on these three days and day 13 were confirmed by real-time PCR. Of the 26,416 equine genes registered, 20,436 transcripts were aligned to sequences in the Ensembl database, from which 4,625 transcripts were registered in both Ensembl and the KEGG pathway. Each of the 4,625 transcripts was examined through KEGG pathway analysis, and 12 transcripts of integrins (*ITG*s) and collagens (*COL*s) were confirmed through real-time PCR. Our data indicated that extracellular matrix (ECM)-related mRNAs were highly expressed in day 19, 21, and 25 conceptus membranes. In combination with previous results, which confirmed a lack of laminin and fibronectin transcript expression in the endometrium, these observations suggest that in contrast to attachment through focal adhesion, conceptus chorionic membrane ECMs function as a scaffold-like structure to possibly maintain the shape of the conceptus and a separation between chorionic membranes and the uterine luminal epithelium.

## Introduction

Among large domestic animal species, the equine conceptus is the slowest to complete implantation and the subsequent placental development [1]. Approximately 12 hours after the equine blastocyst enters the uterus on days 6–6.5 (day 0 = day of ovulation), it is covered with a “capsule” composed of high molecular weight, mucin-like glycoproteins that are rich in threonine and serine residues [2], [3]. The capsule allows the conceptus to transmigrate the entire uterine horns and body over the next 10 days [4]. It is thought that maternal recognition of pregnancy, caused by physical and/or biochemical messages from the conceptus to the maternal endometrium, is required for the prevention of corpus luteum degradation and the continuation of progesterone secretion [5], [6]. In the mare, the first embryonic message, though yet unidentified, is thought to be delivered between days 10 and 14, suppressing the cyclical release of prostaglandin F_2α_ (PGF_2α_) from the endometrium [7]–[10], prolonging the primary corpus luteum life span.

Conceptus movement within the entire uterine tract abruptly ceases on days 16–17, when increases in conceptus diameter and myometrial tone reaches a point where the embryo can no longer pass through the narrow uterine lumen and the conceptus is fixed at the base of either of the uterine horns [11]–[14]. One might expect that after embryo fixation, the conceptus would continue to increase in size; however, ultrasound images have revealed that conceptus sizes are similar between days 17 and 27 [15], during which the heart forms and the capsule disappears. One week after embryo fixation, the capsule dissociates; however, the embryonic vesicle does not appear to firmly attach to the uterine epithelium [1], [16]. Equine embryogenesis is characterized by the development of the chorionic girdle on the outer surface of the chorion between days 25–30 [17]. Although equine conceptuses represent a non-invasive mode of placentation, antigenically foreign trophoblast cells of the chorionic girdle invade into the maternal endometrium on days 36–38, resulting in the formation of endometrial cups [18], [19], from which equine chorionic gonadotropin (eCG) is secreted [20]. Nevertheless, implantation and early placentation in the mare represent a unique variant of mammalian pregnancy [1].

Despite extensive experiments conducted to study pregnancy establishment in the mare, events between the maternal recognition of pregnancy and eCG secretion have not been well characterized. Because conceptus sizes appear to be static even after capsule disintegration [15], it is speculated that other as yet unidentified factors are responsible for conceptus protection during the periods of embryo fixation and capsule disappearance. We hypothesized that extracellular matrix (ECM) and/or cell adhesion molecule (CAM) produced from the conceptus side serves as protection between the start of capsule disintegration and the formation of endometrial cups. Therefore, in this study, we analyzed transcripts of integrins (*ITG*s) and collagens (*COL*s) expressed in the membranes of equine conceptuses during pre- and post-capsule disappearance (days 19, 21, and 25) by RNA-seq (SOLiD3). Several SOLiD3 results were confirmed by real-time PCR with RNAs extracted from days 13, 19, 21, and 25 conceptus membranes. These results were also compared with those of previous uterine endometrium studies in the laboratory [21]–[23].

## Materials and Methods

### Animals and tissue collections

A total of 11 Thoroughbred mares (4–16 yr) were assigned to this study at Hidaka Training and Research Center, JRA (Research Center). The study protocol including sampling procedures and euthanasia was thoroughly reviewed and approved by the Animal Welfare and Ethics Committees at the JRA (approval number; 2007-2, 2008-15, 2009-7) and the University of Tokyo. Horses were allowed to graze together each day and were fed twice daily on a balanced ration of pelleted feed and hay. Ovaries were monitored by rectal palpation and ultrasonography (ECHOPAL, Hitachi, Tokyo, Japan) with a 5.0–7.5 MHz changeable probe (EUP-O33J) [24]. To synchronize estrous cycles, PGF_2α_ (0.25 mg/mare, Planate; Dainippon Sumitomo Pharma Co., Ltd., Osaka, Japan) was injected intramuscularly during the luteal phase. Human chorionic gonadotropin (hCG, 2,500 IU/mare, Gonatropin; Asuka Pharmaceutical Co., Ltd., Tokyo, Japan) was then administered to induce ovulation when growing follicles of over 3.5 cm in diameter were observed.

All mares were initially mated with fertile stallions at the appropriate timing, and ultrasonography was used to confirm the presence of a conceptus. Conceptuses on days 13, 19, 21, and 25 were obtained via uterine flushing with warmed physiological saline (2,000 ml). Approximately 30 days after the uterine flushing to collect a conceptus, 11 mares were again treated for estrous induction, followed by ovulation induction, and nine mares were mated with fertile stallions whereas two control mares were not mated and their uteri collected on day 13 of the estrous cycle. Uteri were then obtained from day 13 cyclic mares (2 mares) and pregnant mares on days 13, 19, 21, and 25 (3, 2, 2, and 2 mares/day, respectively) immediately following slaughter at the Research Center. The uterine horns and body were examined, and conceptuses were carefully collected after longitudinal incision of uterine body and horn. Day 13 conceptuses were immediately frozen, while for day 19, 21, and 25 conceptuses, yolk sac fluid was carefully removed and heart and blood vessel structures were separated from membranes before freezing. The extraembryonic membrane samples consisted of yolk sac and chorion on day 19 and 21 whereas on day 25, the samples contained yolk sac and allantochorion membranes. The membranes and heart/blood vessels, frozen separately in liquid nitrogen and stored at −70°C, were transferred to the Laboratory of Theriogenology and Animal Breeding at the University of Tokyo.

### RNA extraction and preparation for RNA-seq analysis

RNA was extracted from each conceptus membrane (80–100 µg) using Isogen (Nippon gene, Tokyo, Japan) according to the manufacturer’s protocol [25]. In our previous studies of the equine endometrium [21]–[23], increases in uterine transcript levels on day 13 of pregnancy (vs. day 13 of cyclic uterine endometrium) were minimal. Thus, RNA from day 19, 21, and 25 conceptuses was used for RNA-seq (SOLiD3) analysis. A portion of total RNA from day 19, 21, and 25 conceptuses (n = 3 each) was pooled (a total of 120 µg/day) and depleted of rRNA using the Ribominus Eukaryote Kit (Life Technologies, Carlsbad, CA, USA). High-throughput sequencing libraries were prepared according to the SOLiD whole transcriptome library preparation protocol [26], and analysis was performed by Life Technologies-Japan (Tokyo, Japan).

### Mapping reads to the equine genome

Nucleotide sequences identified by RNA-seq analysis were filtered for those containing SOLiD adapters and barcodes. Each read sequence was 50 nt in length, but four nucleotides from the 3′ terminus were excluded for accuracy according to the Applied Biosystems Whole Transcriptome Analysis Pipeline (AB WT Analysis Pipeline, http://www.solidsoftwaretools.com/) protocol. The detailed RNA-seq analysis from this laboratory was described previously [27]. In this pipeline, each read was divided into two 23-base fragments, and the two fragments were mapped to the equine genome (Ensembl: Equus_caballus. EquCab2.55.dna.toplevel.fa). Following standard parameters of AB WT Analysis Pipeline, predicted transcribed regions, novel transcribed regions and annotated transcribed regions were mapped (NTRFinder.75_4.0_0.5_24_0.1.plus.gff). Two mismatches were allowed during mapping, and reads were removed from the analysis if they were aligned to more than 10 locations on a gene. Matching locations were subsequently used to generate counts (read numbers) for the Ensembl-provided coding sequences. Among all mapped transcripts, the transcripts that aligned to the Ensembl equine database and were also found in the Kyoto Encyclopedia of Genes and Genomes (KEGG) pathway analysis (http://www.genome.ad.jp/kegg/pathway.html) [28], [29] were selected for further pathway analysis.

### PCR and qPCR

Based on our hypothesis that conceptuses are covered with ECMs such as COLs and their receptors, ITGs, and are protected even after capsule disappearance, transcripts of *COL*s and *ITG*s were chosen for SOLiD data validation and further analysis. These included 8 collagens (*COL4A1*, *COL4A2*, *COL4A5*, *COL4A6*, *COL5A2*, *COL6A1*, *COL6A2* and *COL6A3*) and 6 integrins (*ITGA3*, *ITGA4*, *ITGA5*, *ITGA6*, *ITGB4* and *ITGB5*). For PCR and real-time PCR analysis, RNA isolated from each conceptus (1 µg each) was reverse-transcribed to cDNA using ReverTra Ace qPCR RT kit (TOYOBO, Osaka, Japan) in a 10 µl reaction volume, and the resulting cDNA (RT template) was stored at 4°C until use. The cDNA reaction mixture was diluted 1∶10 using DNase and RNase-free molecular biology grade water, and 3 µl were used in each amplification reaction. RT template (cDNA) was subjected to PCR or real-time PCR amplification using primers shown in Table 1. Amplification products were separated on 1.5% (w/v) agarose gels after 30 cycles, and PCR products were subcloned and verified by DNA sequencing [30].

**Table 1:** Oligonucleotide primers for RT-PCR and real-time PCR analyses.

Name (GenBank accession No.)	Sequence	Product Length (bp)
*COL4A1* (XM_001496530)	F: 5′- atttcagggaatgccagggg -3′	226
	R: 5′- ttctcccaatggtcctgtgc -3′	
*COL4A5* (XM_001490445)	F: 5′- ctgcttggaggagtttcgtt -3′	133
	R: 5′- cgactgaggtttgctgaaca -3′	
*COL4A6* (XM_001492470)	F: 5′- ctggtctcacctggctcct -3′	130
	R: 5′- gcctctcctccactgttgtc -3′	
*COL5A2* (XM_001501817)	F: 5′- actgggacccctggagatac -3′	214
	R: 5′- ggccaggttcacccttttct -3′	
*COL6A1* (XM_001488351)	F: 5′- agcaagtgtgctgctccttc -3′	190
	R: 5′- cggctccctttttctccctt -3′	
*COL6A2* (XM_001489560)	F: 5′- atggacagaagggcaagctg -3′	219
	R: 5′- ctggggatccattgttgcct -3′	
*COL6A3* (XM_003364191)	F: 5′- ttggagcattggaaaggaac -3′	134
	R: 5′- ggaactcggtatgtgggttg -3′	
*ITGA4* (XM_001917601)	F: 5′- ccgcctgtgaaaatgaaggg -3′	152
	R: 5′- tccaggctcattttcctccg -3′	
*ITGA5* (XM_001504571)	F: 5′- ttcaacttagacgcggaggc -3′	239
	R: 5′- aggatccgagaacctttgctg -3′	
*ITGA6* (XM_001495066)	F: 5′- cggtctccggagttgctaaa -3′	154
	R: 5′- gccgtgccgaggtttttaag -3′	
*ITGB4* (XM_001915880)	F: 5′- tgcacaggaagaaggactgc -3′	231
	R: 5′- gtgtgtccaggtggtctgag -3′	
*ITGB5* (XM_001500027)	F: 5′- cctgaatgaggccaacgagt -3′	236
	R: 5′- accggatgctattgtacgca -3′	
*GAPDH* (NM_001163856)	F: 5′- catcctgggctacactgagg -3′	163
	R: 5′- gtccaccaccctattgctgt -3′	

F: Forward, R: Reverse.

Quantitative PCR reactions were performed with the SYBR Green kit (Takara Biomedicals, Tokyo, Japan) and the Applied Biosystems thermal cycle system (7900HT, Applied Biosystems, Tokyo, Japan) as previously described [31]. The amplification efficiencies of each target gene and the reference gene *GAPDH* were examined through calibration curves and found to be comparable [32]. PCR amplification consisted of 40 cycles at 95°C for 10 s, annealing at 60°C for 20 s, and extension at 72°C for 30 s. The threshold cycle (Ct) value for each target was determined by Sequence Detection System software v1.2 (Applied Biosystems). The expression levels of each mRNA were normalized by subtracting the Ct value of the target mRNA from the Ct value of the internal control, *GAPDH* mRNA. Each amplification was completed with a melting curve analysis to confirm the specificity of amplification and absence of primer dimers [31].

## Results

### Numbers of recovered conceptuses

From the initial mating of 11 mares with fertile stallions, the numbers of conceptuses collected from the uterine flushing procedure were 8, 4, 2, and 2 for days 13, 19, 21, and 25, respectively. All mares were subsequently subjected to the ovulation treatment, and nine out of 11 mares were mated with fertile stallions. These mares were slaughtered and uteri removed, from which 3, 3, 2, and 2 conceptuses were carefully removed from day 13, 19, 21, and 25 pregnant mares, respectively. These procedures resulted in the collection of 11, 7, 4, and 4 conceptuses from day 13, 19, 21, and 25 pregnant mares, respectively.

### RNA-seq analysis and validation through qPCR analysis

We mapped short reads from the Whole Transcriptome Analysis Pipeline (Applied Biosystems-Life Technologies, Grand Island, NY, USA) for those identified in day 19, 21, and 25 conceptuses onto the equine genome. Among 26,416 equine genes registered, 20,436 transcripts identified were aligned to the Ensembl database; of those, 4,625 transcripts were found in both Ensembl and KEGG pathway [28], [29]. Each of the 4,625 transcripts was examined for their read counts, following the Whole Transcriptome Analysis Pipeline protocol [33]. In these analyses, transcript changes between days 19 and 21 and days 21 and 25 were divided into up/up, up/down, down/up, and down/down (S1 Table). Transcripts that were unchanged were categorized as down-regulated genes.

**Figure 1:**
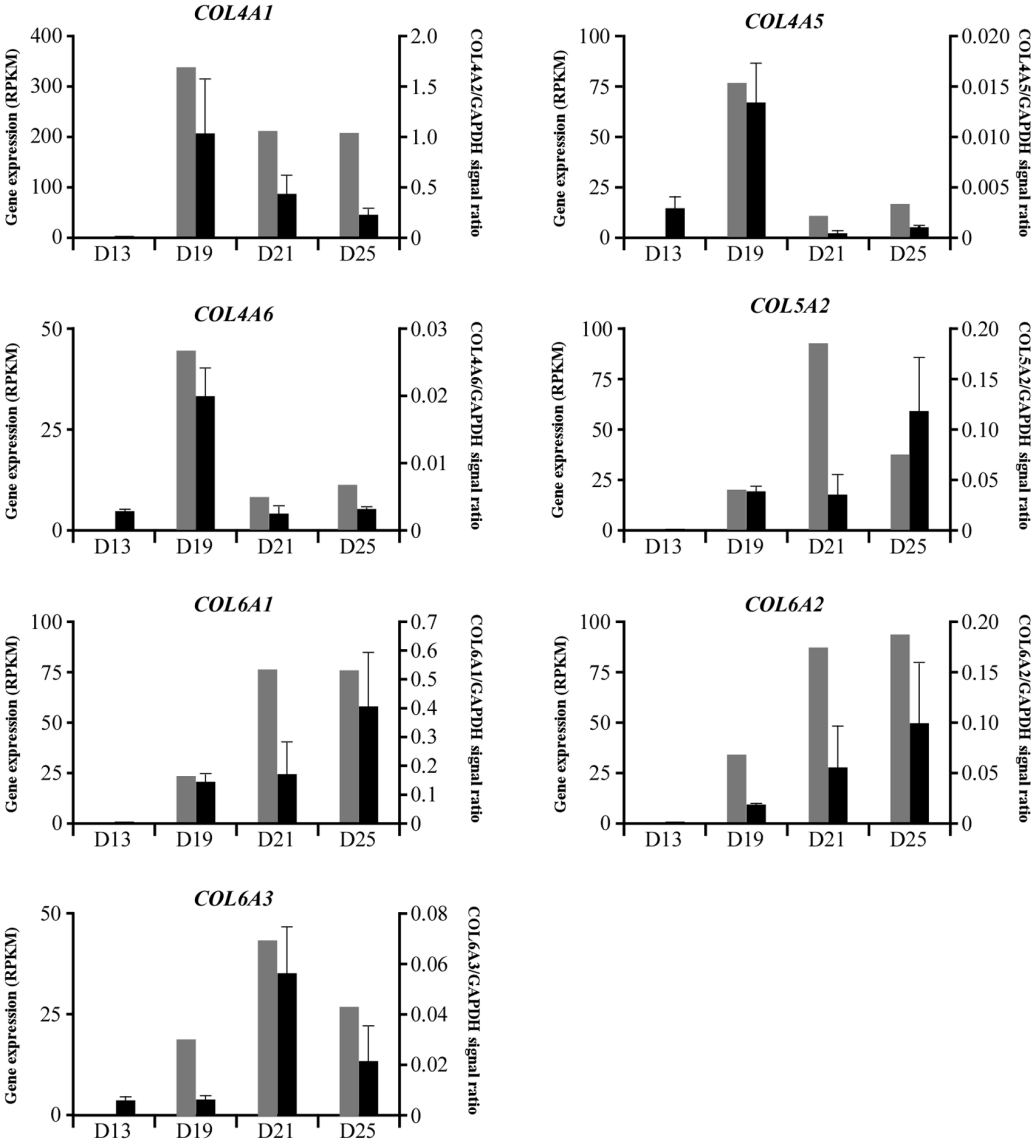
Collagen transcript analysis by qPCR vs. RNA-seq. Total RNA was isolated from whole day 13 conceptuses, while for day 19, 21, and 25 conceptuses, yolk sac fluid was carefully removed and heart and blood vessel structures were separated from extraembryonic membranes. The extraembryonic membrane samples consisted of yolk sac and chorion on day 19 and 21 whereas on day 25, the samples contained yolk sac and allantochorion membranes. Collagen transcripts analyzed were *COL4A1*, *COL4A5*, *COL4A6*, *COL5A2*, *COL6A1*, *COL6A2*, and *COL6A3*. Gray bars indicate data from RNA-seq analysis, and dark bars represent data from qPCR analyses. Note that RNA-seq data represent transcripts extracted from day 19, 21 and 25 trophectoderm and/or chorionic membrane, whereas RNA from days 13, 19, 21 and 25 were analyzed by qPCR. An average of at least three conceptuses was analyzed for each time point, and the SEM is shown. Please note that values on Y-axis resulted from the generation of a standard curve for each transcript. In doing so, the highest amount in each transcript was treated as “1”, and relative values were presented.

**Figure 2:**
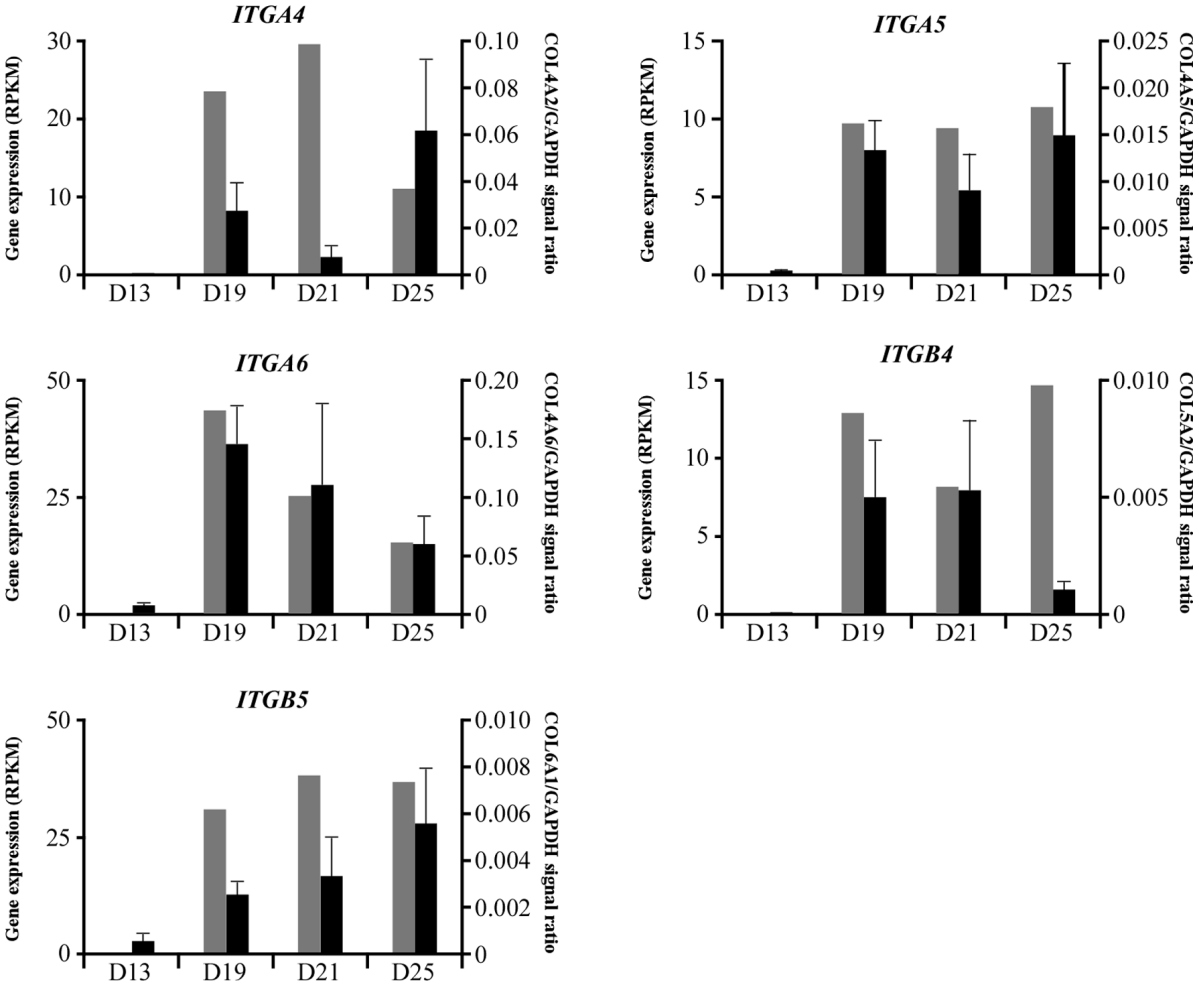
Changes in integrin transcripts, qPCR vs. RNA-seq. Total RNA was isolated from whole day 13 conceptuses, while for day 19, 21, and 25 conceptuses, yolk sac fluid was carefully removed and heart and blood vessel structures were separated from extraembryonic membranes. The extraembryonic membrane samples consisted of yolk sac and chorion on day 19 and 21 whereas on day 25, the samples contained yolk sac and allantochorion membranes. Integrin transcripts analyzed were *ITGA4*, *ITGA5*, *ITGA6*, *ITGB4* and *ITGB5*. Gray bars indicate data from RNA-seq analysis, and dark bars represent data from qPCR analyses. Note that RNA-seq data represent transcripts extracted from day 19, 21 and 25 trophectoderm and/or chorionic membrane, whereas RNA from days 13, 19, 21 and 25 were analyzed by qPCR. An average of at least three conceptuses was analyzed for each time point, and the SEM is shown. Please note that values on Y-axis resulted from the generation of a standard curve for each transcript. In doing so, the highest amount in each transcript was treated as “1”, and relative values were presented.

From the RNA-seq analysis, extensive expression of *COL* and *ITG* transcripts was found. To confirm the data from RNA-seq analysis, quantitative PCR (qPCR) analyses on *COL* and *ITG* transcripts in day 13, 19, 21, and 25 conceptuses were conducted to determine transcript levels. Transcripts examined were *COL4A1*, *COL4A2*, *COL4A5*, *COL4A6*, *COL5A2*, *COL6A1*, *COL6A2*, and *COL6A3* (Fig. 1), and *ITGA3*, *ITGA4*, *ITGA5*, *ITGA6*, *ITGB4*, and *ITGB5* (Fig. 2). Two transcripts, *COL4A2* and *ITGA3*, could not be amplified at the stage of primer validation. However, the changes in 7 *COL* and 5 *ITG* transcripts were determined through the use of RNA-seq and qPCR analyses. Previous studies [21]–[23] have indicated that transcripts related to laminin or fibronectin were not found on the endometrial side during the same period.

### RNA-seq analysis of transcripts involved in cell-cell interactions

**Figure 3:**
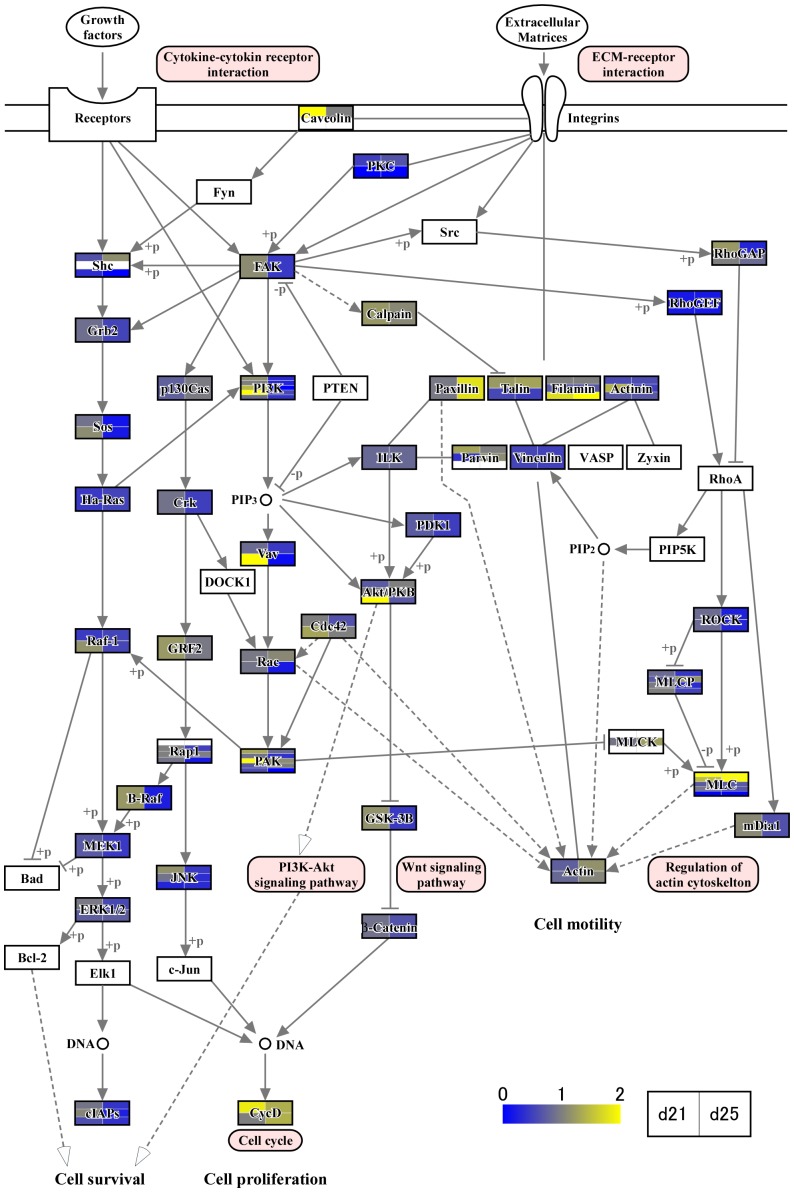
Focal adhesion pathway analysis. Identified transcripts were aligned to the Ensembl equine database and analyzed through the Kyoto Encyclopedia of Genes and Genomes (KEGG) pathway analysis (http://www.genome.ad.jp/kegg/pathway.html). Because we expected that scaffold-like structures form during these time points, the first KEGG analysis examined focal adhesion; cytokine-cytokine interactions and ECM-receptor interactions. Data represent transcripts of day 21 and 25 conceptuses. There are more than two layers, i.e., Caveolin transcripts were found in the cell membrane on top [43]. Yellow color on the top-left side of box indicates the transcript of one of the Caveolin variants increased on day 21 (vs. day 19). White color on the left and right of the bottom indicates that the transcripts related to the second Caveolin variant were not found on days 21 or 25 in this RNA-seq analysis.

**Figure 4:**
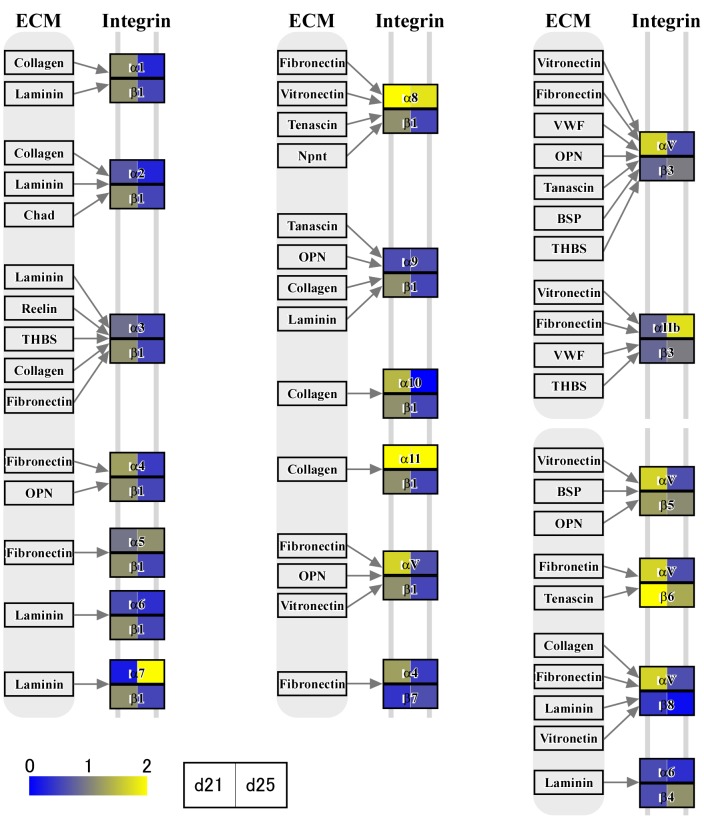
ECM receptor pathway analysis; integrin transcripts. Pathway analysis was extended to the expression of integrin transcripts. There are three columns, each containing potential ligands and integrin receptor heterodimers. αVβ3 is on the upper right: αV transcripts were high on day 21 and decreased toward day 25.

**Figure 5:**
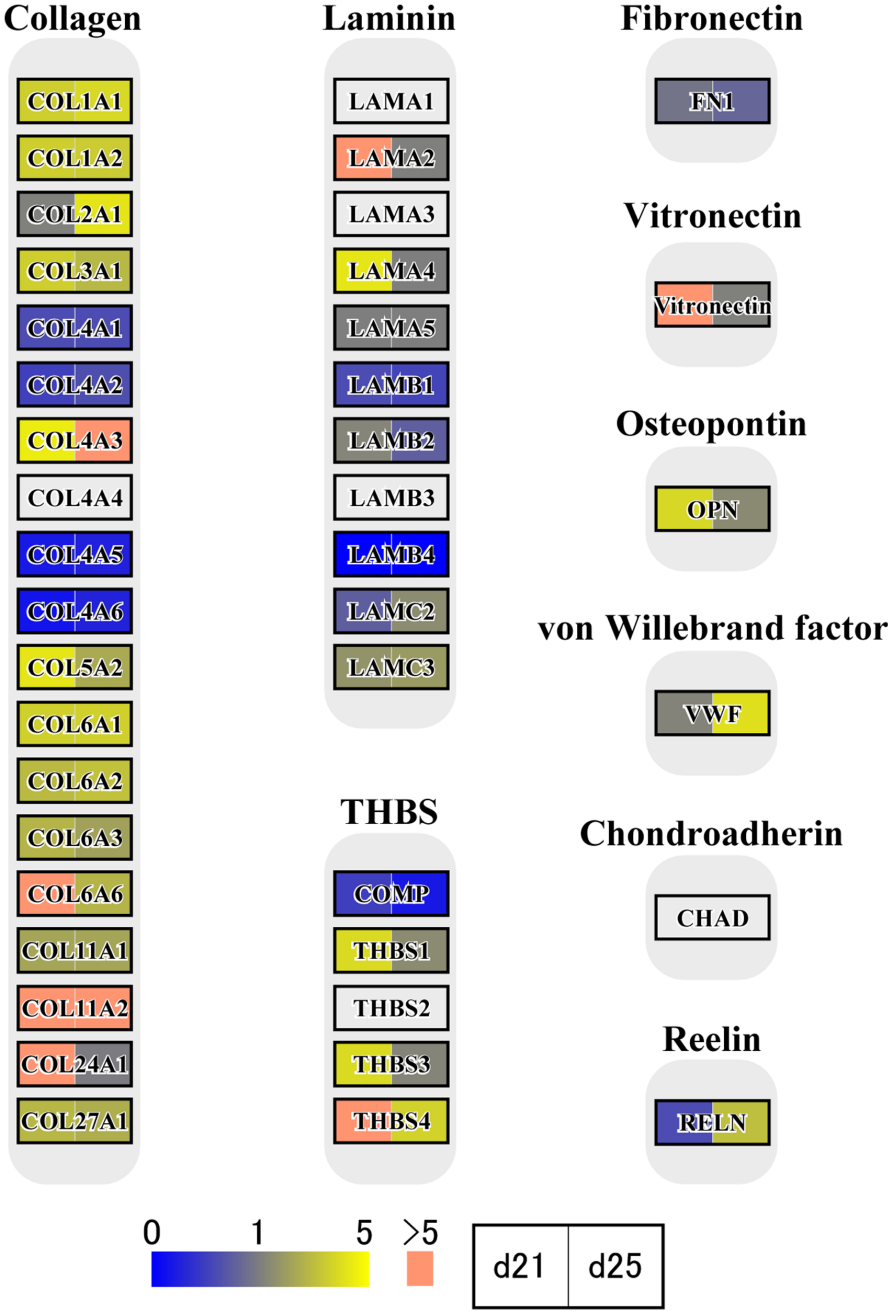
ECM receptor pathway analysis; collagen, laminin, fibronectin, vitronectin, osteopontin, von Willebrand factor, chondroadherin, and reelin transcripts. Pathway analysis was further extended to the expression of collagen, laminin, fibronectin, vitronectin, osteopontin, von Willebrand factor, chondroadherin, and reelin transcripts. *COL4A3* transcripts are on the left in yellow and on the right in pink, indicating that *COL4A3* expression is nearly 5-fold higher on day 21 than day 19, and further increased on day 25.

**Figure 6:**
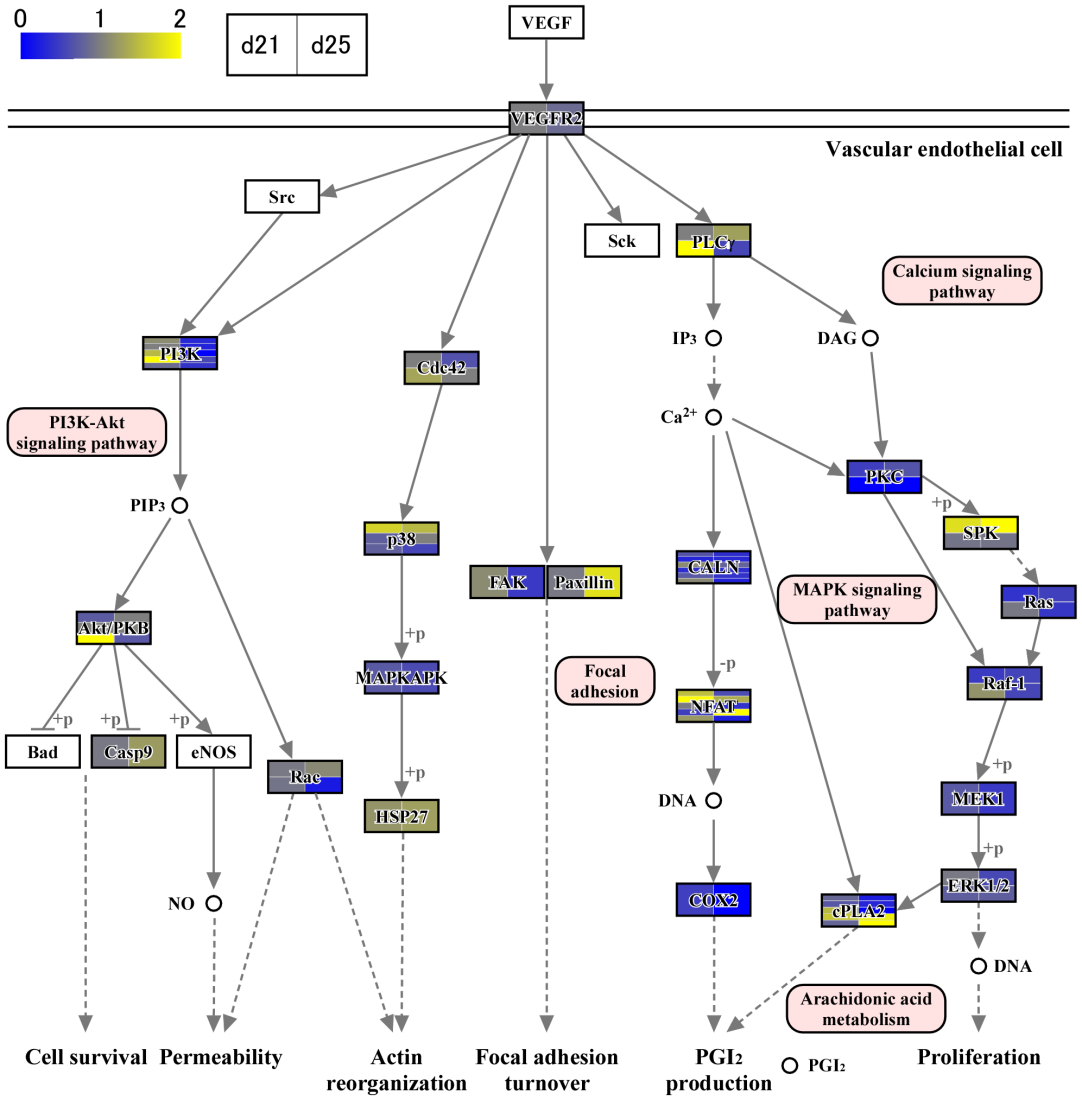
VEGF signaling pathway analysis. Because heart and vessel structures appear and dynamic membrane development occurs during this time period, KEGG pathway analysis was extended to study VEGF signaling pathway [40], [41]. Events downstream of VEGF-VEGFR2, such as actin reorganization and focal adhesion turnover, were activated on day 25.

In the previous studies [21]–[23], [34], a substantial number of growth factors and cytokine expression, including vascular endothelial growth factor (VEGF) [S. Haneda, unpublished observations], are found in the endometrium. In this study, we identified numerous transcripts through RNA-seq analysis. To characterize transcript expression of conceptus membranes related to cellular processes, groups of genes associated with focal adhesions (Fig. 3), cytokine-cytokine receptor interaction, chemokine signaling pathway, CAMs, and ECM-receptor interactions (Figs. 4 and 5) were characterized through KEGG pathway analyses. During this time period of conceptus development, heart and vascular formation take place, the latter of which is also expected in yolk sac and allantochorion membranes. Transcripts found in conceptus membranes were then aligned to those related to the VEGF signaling pathway (Fig. 6). Among days 19, 21 and 25, the highest expression of VEGF-related pathways was found on day 21, concurrent with capsule disintegration.

## Discussion

Following intra-uterine migration, embryo fixation occurs on days 17–18. However, the conceptus remains covered with a capsule, which starts to dissociate on days 22–23 [4], [35], although significant portions of the capsule persist on conceptus membranes until as late as day 32 of gestation [36]. One might expect that the disappearance of the capsule would then be followed by the formation of a tight connection between the trophectoderm-chorionic membrane and uterine epithelium. However, collection of the whole conceptus by uterine flushing is still possible until days 35–36 of pregnancy [1], [37], which implies that the trophectoderm and uterine epithelial cells have not yet fully adhered. Based on the observed increase in transcripts related to ECM components in day 19, 21, and 25 conceptus membranes in this study and on the endometrial components detected in previous studies [21]–[23], we propose that the chorionic membrane and uterine epithelium do not adhere after capsule disintegration; rather, the conceptus side builds scaffold-like structures on the outer membranes, which expand into gaps in the deteriorating capsule, preserving a degree of separation between the two cell types, while still allowing the conceptus to benefit from close proximity to the uterine epithelium and its secretions.

The capsule plays an important role in intra-uterine migration of the equine conceptus. It is thought that embryo fixation on days 17–18 results from the increase in endometrial tone and the conceptus reaching its maximum size within the capsule [38], restricting its movement within the uterine lumen. Conceptus growth appears to pause at this point, and the capsule may take 4 or 5 days to disintegrate; however, even freed from the capsule, conceptus size is static until chorionic girdle formation is initiated [39]. The maintenance of conceptus size for several days after capsule disappearance indicates that the capsule itself may not be the sole limiting factor for conceptus size. During this time period, dynamic development of heart, vessel and allantochorion formation takes place. Endometrial cytokine and growth factor expression appear to stimulate conceptus membranes, resulting in the activation of PI3K, AKT and Wnt intracellular signaling pathways [40], actin cytoskeleton regulation and cell adhesion molecule expression (Figs. 3, 4 and 5). The VEGF signaling system also appears to be activated in conceptus membranes [40], possibly resulting in the onset of focal adhesion, PI3K-AKT and MAPK intracellular signaling pathways [41] as well as focal adhesion turnover and arachidonic acid metabolism (Fig. 6). Although these findings require further study for their confirmation, gene expression in the outer membranes of equine conceptuses during conceptus fixation and capsule disappearance is more dynamically regulated than is generally assumed to facilitate extraembryonic membrane development.

The capsule helps maintain the tertiary structures of the conceptus and protects the conceptus from the maternal uterine environment. The capsule may serve as a barrier against the maternal immune system while maintaining biochemical communication between the conceptus and the uterine endometrium. Notably, as embryo fixation occurs, immune cells migrate into the uterine endometrium toward the luminal epithelium where the embryo is lodged [36]. This observation and our previous results [21], [23] suggest that immunological responses from the endometrial side are present even before the trophectoderm-chorionic membrane attaches to the uterine epithelium. The construction of scaffold-like structures on the conceptus side could prevent direct contact between the developing conceptus and endometrial epithelial cells, and possibly immune cells, minimizing the immune response against the conceptus.

Conceptuses can be collected by uterine flushing until days 35–36, suggesting that the unencapsulated conceptus is only loosely attached to the uterine epithelium until the period of endometrial cup formation and eCG production [37]. In our previous cDNA subtraction analyses [21]–[23], endometrial *eCG* mRNA expression was not detected on day 25, indicating that chorionic girdle and/or endometrial cup formation had not yet occurred. In addition, the genes related to *COL*s and *ITG*s were most highly expressed in day 19, 21, and 25 conceptuses. In our previous studies [21]–[23], the highest gene expressions from the endometrial side were found on days 19–21. It should be noted that transcripts for laminin or fibronectin genes were not detected in day 13, 19, 21, or 25 endometrial tissues. These results indicate, firstly, that although the conceptus membrane expresses *COL* and *ITG* transcripts, there are no binding partners on the uterine epithelium; and secondly, that the disappearance of the equine capsule does not trigger trophectoderm adhesion to the uterine epithelium and/or the generation of chorionic girdle. These observations suggest that ECM scaffolding [42] with COLs/ITGs and other ECM components not yet identified maintains the conceptus structure and possibly its size while the conceptus is lodged but not directly attached to the uterine epithelium.

## Conclusion

This study represents the first study utilizing a next generation sequencer to identify transcripts found in equine conceptus membranes during embryo fixation and capsule disappearance. Based on our confirmation of numerous transcripts related to trophoblast/uterine epithelium interactions, we propose that as the capsule disintegrates, ECM scaffolding develops from the conceptus membrane, preventing direct contact with the maternal tissues and avoiding the immune response until eCG production begins and pregnancy can be established.

## Supporting Information

S1 Table **Transcript changes in day 19, 21, and 25 equine conceptuses.** Transcripts on days 19, 21, and 25 are summarized by functional groups and fold-changes.
(XLSX)
